# Dose modification factor analysis of multilumen balloon brachytherapy applicator with Monte Carlo simulation

**DOI:** 10.1120/jacmp.v15i3.4498

**Published:** 2013-05-08

**Authors:** David Pearson, Eric A. Williams

**Affiliations:** ^1^ Department of Radiation Oncology University of Toledo Health Science Campus Toledo OH USA

**Keywords:** Monte Carlo, HDR, multilumen applicator, brachytherapy, partial breast irradiation

## Abstract

The Contura brachytherapy applicator is a silicone balloon with five lumens in which a high‐dose‐rate brachytherapy source can traverse. Multilumen applicators, like the Contura, are used in accelerated partial breast irradiation (APBI) brachytherapy in instances where asymmetric dose distributions are desired; for example, when the applicator surface‐to‐skin thickness is small (< 7 mm). In these instances, the air outside the patient and the lung act as a poor scattering medium, scattering less dose back into the breast and affecting the dose distribution. The recent report by Task Group 186 of the American Association of Physicists in Medicine (AAPM) has outlined the importance of moving towards brachytherapy dose calculations using heterogeneity corrections. However, at this time, many commercial treatment planning systems do not correct for tissue heterogeneity, which can result in inaccuracies in the planned dose distribution. To quantify the deviation in the skin dose we utilize the dose modification factor (DMF), defined as the ratio of the dose rate at 1 cm beyond the applicator surface with homogenous medium, to the dose rate at 1 cm with heterogeneous medium. This investigation models the Contura applicator with the Monte Carlo N‐Particle code version 5, and determines a DMF through simulation. Taking all geometrical considerations into account, an accurate model of the Contura balloon applicator was created in MCNP and used to run simulations. The dose modification factor was found to be only slightly dependent on whether the dose distribution was symmetric or asymmetric. These results indicate that the dose delivered to part of the PTV may be lower than the planned dose by up to 12%, and that these brachytherapy plans should be viewed with caution. In addition to studying the effects of backscatter, an evaluation was made regarding the capabilities of the Contura device to shape an asymmetric dose distribution. We compared these results to a previous study of a MammoSite ML and a SAVI device and found that the dose shaping capabilities of the Contura were quite similar to that of the MammoSite ML, but markedly inferior to the SAVI.

PACS number: 87.53.Jw

## I. INTRODUCTION

Accelerated partial breast irradiation (APBI) was developed as an alternative to whole‐breast irradiation for early‐stage breast cancer, and has rapidly become a widely used option.^(1)^ The goal of APBI is to spare normal tissue in the breast and treat only a margin around the lumpectomy cavity, where cancer is most likely to recur. This can be accomplished using high‐dose‐rate brachytherapy (HDR). The typical APBI treatment course utilizing HDR lasts five days, with treatments occurring twice a day separated by at least 6 hours. This reduces the total radiation course from six to seven weeks to one week, which can make it a more convenient option for those who qualify. The prescription dose, as defined in the NSABP B‐39/RTOG 0413 randomized phase III trial,^(2)^ is 340 cGy per treatment, totaling 3400 cGy over ten treatments, given to the PTV which is delineated with 1 cm expansion of the balloon in lumpectomy cavity, assuming no organs at risk encroach on this volume.

A major drawback in many of the brachytherapy treatment planning systems currently in use is that inhomogeneity corrections are not taken into account. Typically, these systems utilize the dose calculations of AAPM TG‐43.^(3)^ The 2012 report by the American Association of Physicists in Medicine (AAPM) Task Group 186^(4)^ states that such corrections should be implemented when possible. With these recommendations in place it seems likely that there will be more widespread use of inhomogeneity corrections in the future. One example of the need for accurate calculations is based on the studies of the contrast solution used in a MammoSite balloon. Various concentrations of contrast solution where used in an investigation to study the effect this would have on the dose delivered to the PTV around the applicator.^5^, ^6^, ^7^, ^8^ For some treatment sites (for example, the treatment of the vaginal mucosa with a vaginal cylinder), the lack of inhomogeneity corrections in the treatment planning algorithm may not be a major concern because there may be no air or bone within the volume being treated. In such cases, assuming that all tissue has a density equivalent to water $\left(1\;g/{\text{cm}}^{3}\right)$ is a reasonable approximation and mimics the assumptions made in the TG‐43 protocol. However, for treatments like APBI where the lumpectomy cavity is close to the skin surface, not correcting for the lack of backscatter from air can cause significant differences in the treatment plan.^(9)^ The difference between the planning system and reality can lead to a plan being created that delivers a lower dose to the treatment volume. This effect has been studied for the MammoSite balloon by Kassas et al. using Monte Carlo simulations^(9)^ and with TLD measurements.^(8)^ Kassas and colleagues created Monte‐Carlo simulations in order to study DMF using a single lumen MammoSite applicator. The balloon was in a spherical water phantom and a single dwell position at the center of the balloon was used. Simulations with varying amounts of backscatter were run by altering the water phantom's radius, and were compared to a simulation with 30 cm of water beyond the measurement point (full backscatter). For small tissue thicknesses, the Kassas study found that differences in planned and delivered dose can be substantial, up to 13% for the largest balloon size and no backscatter material beyond the dose calculation point.

More recently, APBI treatment devices capable of asymmetric dose distributions have been introduced. These devices, such as the SAVI (Cianna Medical, Inc., Aliso Viejo, CA), Contura (SenoRx, Inc., Irvine, CA) and the MammoSite ML (Hologic, Inc., Marlborough, MA) offer this advantage by using multiple lumens within the device. By more heavily weighting source dwell positions on one side of the device, it is possible to steer the dose away from critical structures. The effect of the lack of tissue for full backscatter for devices capable of asymmetric dose distributions, such as the Contura, has not been studied in detail.^10^, ^11^, ^12^

In this work we investigate the effect of having limited amounts of tissue beyond the surface of the Contura balloon applicator. We quantify this effect with a dose modification factor, which is simply a ratio of the dose to the PTV surface with and without full backscatter, where full backscatter is the situation with an infinite amount of material beyond the dose calculation point. We show how capable the Contura applicator is at producing asymmetric dose distributions, when compared to the MammoSite ML and the SAVI applicators. We also present the results of our study on how this factor changes with the use of asymmetry in the dose distribution.

## II. MATERIALS AND METHODS

### A. Asymmetry study of the Contura applicator

The Contura multilumen applicator has four lumens surrounding a central lumen, each separated radially by 90°. The maximum distance between the central and outer lumens is 5 mm, occurring at the center of the balloon. The Contura is available in two sizes: 4–5 cm and 4.5–6 cm diameters. These sizes enable the Contura to fill to the same volumes as single‐lumen applicators.

The advantage of multilumen applicators is their ability to create an asymmetric dose distribution. The amount of asymmetry obtainable from an applicator can be quantified by using points around the applicator and quantifying the difference in the doses at these points. Four reference points were placed at cardinal directions 1 cm from the balloon surface on an axial plane where the balloon diameter was widest. A symmetric plan would give the same dose to each of these points. To create an asymmetric plan, three of these points were held constant (within 1%) while reducing the dose to the remaining point as much as possible. This method was chosen to best match a clinical situation where the dose to the planning target volume (PTV) would be kept constant while trying to spare a critical structure, such as the skin. The percentage difference between the dose at the asymmetric point to the average of the other three points is then the maximum asymmetry the particular applicator can achieve.

### B. MONTE CARLO study of the dose modification factor

**Figure 1:**
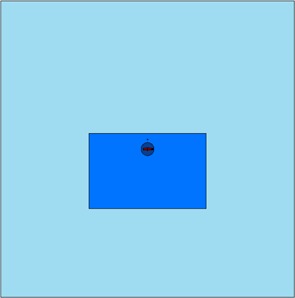
Contura model in cuboid water phantom with 2 cm of backscatter.

The software used for these simulations was the Monte Carlo N‐Particle Transport Code MCNP5 (Los Alamos National Laboratory, Los Alamos, NM). MCNP5 is a general‐purpose, continuous‐energy code capable of simulating neutron, photon, and electron transport.^(13)^ The Contura model consisted of a sphere with a radius of 2.2 cm to represent the balloon. Inside the balloon, 35 right circular cylinders were created to simulate the dwell positions of the applicator. The source used in this study was the VS2000 used in Varian iX HDR afterloaders (Varian Medical Systems, Palo Alto, CA). Each source was 0.5 cm long with a radius of 0.017 cm and composed of pure ^192^Ir. The photon energy spectrum of ^192^Ir was taken from U.S. Department of Energy Radioactive Decay Tables.^(14)^ A small sphere with radius 0.17 cm was used as a measurement volume, simulating the ion chamber used in previous physical measurements. The BOX macrobody was used to create a cuboid water phantom, as seen in Fig. 1.

**Figure 2:**
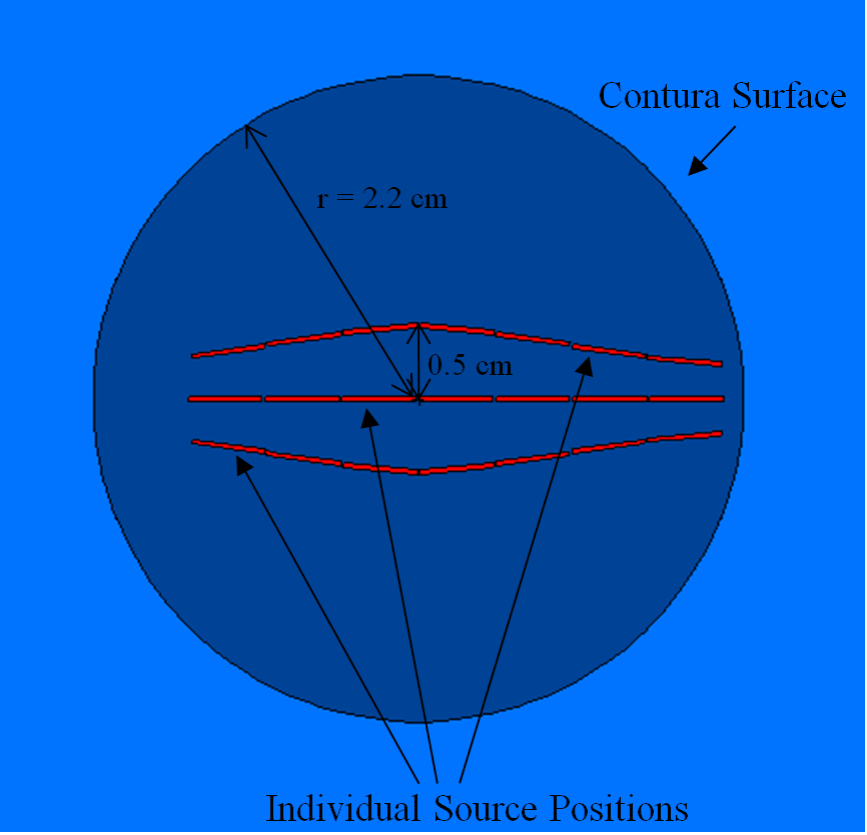
Modeled Contura applicator geometry.

The cuboid water phantom measured 40 cm by × by 40 cm, with × varying from 23.2 cm to 33.2 cm to simulate a range of tissue depths. The Contura applicator model was placed in the center of the phantom in the X and Z planes. This phantom geometry was chosen in order to compare our simulations to measurements previously taken.^(15)^ Everything within this box, except the sources, was set to a density of water. This ignores the thin silicone wall of the balloon and nylon catheters, which were assumed to have negligible effect on the dose. Outside of this box, a cube with sides 100 cm in length was used as air and to form a boundary for the simulation. A spherical phantom was also created in order to study a more anthropomorphic circumstance. A sphere was created with radius varying from 3.2 cm to 13.2 cm to achieve the same depths as the cuboid phantom. This phantom was also surrounded by a $100\;{\text{cm}}^{3}$ cube of air used as a boundary. A cross‐sectional view of the modeled geometry of the Contura applicator is shown in Fig. 2.

Each simulation was adjusted to minimize the uncertainty in measurements and to pass all of the statistical checks within MCNP. This was accomplished mainly by increasing the number of particles in the simulation. $5\times 10^{7}$ particle histories were used for each simulation, which gave uncertainties ≪0.5%.

### C. Treatment plans

Symmetric and asymmetric dose distributions for the Contura applicator were created using the BrachyVision 10.0 (Varian Medical Systems) treatment planning system. By default, this system computes dose using the formulism developed by AAPM Task Group 43^(3)^ which assumes a homogeneous water medium. BrachyVision calculates the dose both inside the patient and beyond the skin boundary in this way. A forward planning technique was used in the BrachyVision treatment planning system along with a dose grid size and resolution of 40×40×40cm and 0.25 cm, respectively. A CT scan was taken of the Contura in a water phantom in order to make the plans. The physical phantom used was 30.5×38×38cm, but it should be noted that these dimensions are academic since the planning system considers everything inside and outside of the phantom to be water. For both the symmetric and asymmetric dose distributions, the five lumens were traced using the tools provided in BrachyVision. Each of the five lumens was filled with seven dwell positions, each of 5 mm dwell step size. Seven dwell positions where chosen to fill each lumen to the maximum, without having a dwell position partially outside the applicator. The dose was then normalized to the points located 1 cm from the surface of the applicator to represent the PTV. In the symmetric case, all four points receive the same dose. For both symmetric and asymmetric cases, the dose distribution was optimized manually (i.e., forward‐planned). The difference between these two plans was that the asymmetric plan did not use the central lumen or the lumen nearest the measurement volume. Following the removal of these source positions, the dose distribution for the asymmetric plan was renormalized to prescription dose. The plan was created in this way to maximize the asymmetry, giving full dose to the PTV in all but the asymmetric direction. The asymmetric dose distribution resulted in a plan where three of the four previously mentioned reference points received full prescription dose (to within 1%), while the dose at the remaining point was reduced as much as possible. The same Monte Carlo model of the Contura was used in each instance, with the weighting factors being changed in order to simulate each plan. In the asymmetric plan, the central lumen and the lumen nearest the measurement point were not used. The dwell times from the treatment plans were then normalized to produce a weighting factor for each dwell position in the plan. When simulations were run, each dwell position produced the same number of particles and the outcome was multiplied by the weighting factor. This made the simulated plan an accurate duplication of the plan in the treatment planning system.

## III. RESULTS & DISCUSSION

### A. Acceptance of the Contura MC model

One goal of this study was to quantify the difference between the BrachyVision‐calculated dose and the MCNP‐calculated dose for different amounts of backscatter present for the Contura multilumen APBI applicator. This was accomplished by creating MCNP simulations and calculating the dose 1 cm from the balloon surface with different amounts of backscatter present beyond the point of measurement, varying from 1 cm to 10 cm increments of 1 cm. These data were compared to simulations performed with full backscatter, emulating current treatment planning systems. From these data, a dose modification factor (DMF) was produced, defined as the ratio of the dose rate at the typical prescription distance of 1 cm from the balloon's surface with full scatter, obtained using a water phantom, to the dose rate with a finite tissue thickness beyond the prescription line. The DMF is essentially the ratio of planned versus delivered dose when using a treatment planning system that does not take into account tissue heterogeneity.

**Figure 3:**
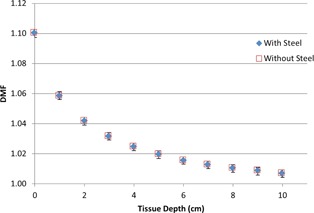
Effect of steel wire on DMF.

The initial model created in this study was based on physical measurements taken using a Contura applicator and a traditional cuboid water phantom. It is shown in Fig. 3 that the wire that the source is contained within does not present significant attenuation and, thus, was not included in the simulations. Since all DMF values were relative, the small amount of attenuation from the wire was found to be negligible.

The largest difference in DMF between the simulations with and without the encapsulating steel wire was found to be −0.55%. The difference in the absolute dose from the two simulations was slightly higher but since all measurements are normalized to the full backscatter point, this effect is minimized. From these results it was concluded that disregarding the wire was reasonable and would be done on all subsequent Contura models.

### B. Asymmetry study of the Contura applicator

**Figure 4:**
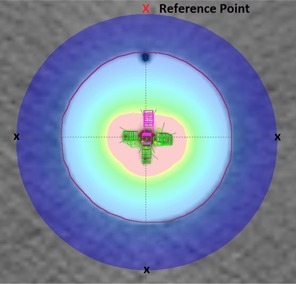
Contura asymmetric dose distribution. Each point used in the simulations is marked by an ‘X’. Three of these points were held at a constant dose. The difference between the reference point and these three points quantifies the dose asymmetry of the plan.

The ability of the Contura to produce an asymmetric dose distribution can be quantified by considering the dose to four points at cardinal positions at a distance of 1 cm from the balloon surface. To simulate a clinical situation, three of the four points were maintained at a prescription dose to within 1%. The fourth, referred to as the reference point, was reduced to as low a dose as possible. An asymmetric dose distribution produced in BrachyVision is shown in Fig. 4.

The dose asymmetry was defined as the percent difference between the dose of the asymmetry point and the average of the remaining three points held at the prescription dose. RT0G0413 provides parameters that should be met for the volumes of tissue receiving 150% (V150) and 200% (V200) of the prescribed dose. These volumes should be limited to ≤50cc and ≤10cc, respectively.^(2)^ The maximum dose asymmetry of Contura applicator was found to be 14.7% with a V150 of $27.9\;{\text{cm}}^{3}$, and a V200 of $8.6\;{\text{cm}}^{3}$.^(15)^ In a previous study,^(16)^ the maximum dose asymmetry for the SAVI and MammoSite ML applicators was measured. The SAVI produced an asymmetry of 37.3% with a V150 of $25.6\;{\text{cm}}^{3}$ and a V200 of $9.69\;{\text{cm}}^{3}$, while the MammoSite ML produced an asymmetry of 12.2% with a V150 of $35.7\;{\text{cm}}^{3}$ and a V200 of $10\;{\text{cm}}^{3}$. Greater asymmetry could be produced, but this would have pushed the V150 and V200 outside the accepted tolerances of RT0G0413. The V200 was found to be the limiting factor. These results are due to the design of the lumens in each of the applicator. In order to steer the dose from one side of the device to the other, the spacing between the lumens in this direction needs to be maximized for greatest effect. The MammoSite ML has the smallest lumen spacing across the internal diameter, followed closely by the Contura, with a maximum spacing of 5 mm between the central and outer lumens. Due to the strut design of the SAVI, the distance from lumens on one side to the other is much greater, and thus the ability to steer the dose for this device is significantly greater. The amount of curvature of the SAVI is designed to be variable.

### C. Effect of symmetry on the DMF

**Figure 5:**
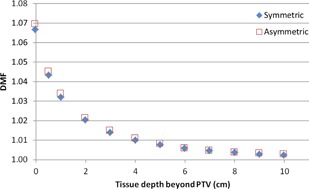
Symmetric vs. asymmetric DMF.

We had considered the possibility that the different dose distributions would cause small spectral changes and scatter differences in the direction of the reference point, but Fig. 5 shows that these effects were not evident. Small deviations in Fig. 5 (<0.3%) were within the statistical uncertainty of the simulations.

There is no significant difference in DMF between symmetric and asymmetric simulations. Since all simulation results are relative to full backscatter data, large differences to the absolute dose at the measurement point are not seen in the DMF.

### D. DMF measurements in different geometries

**Figure 6:**
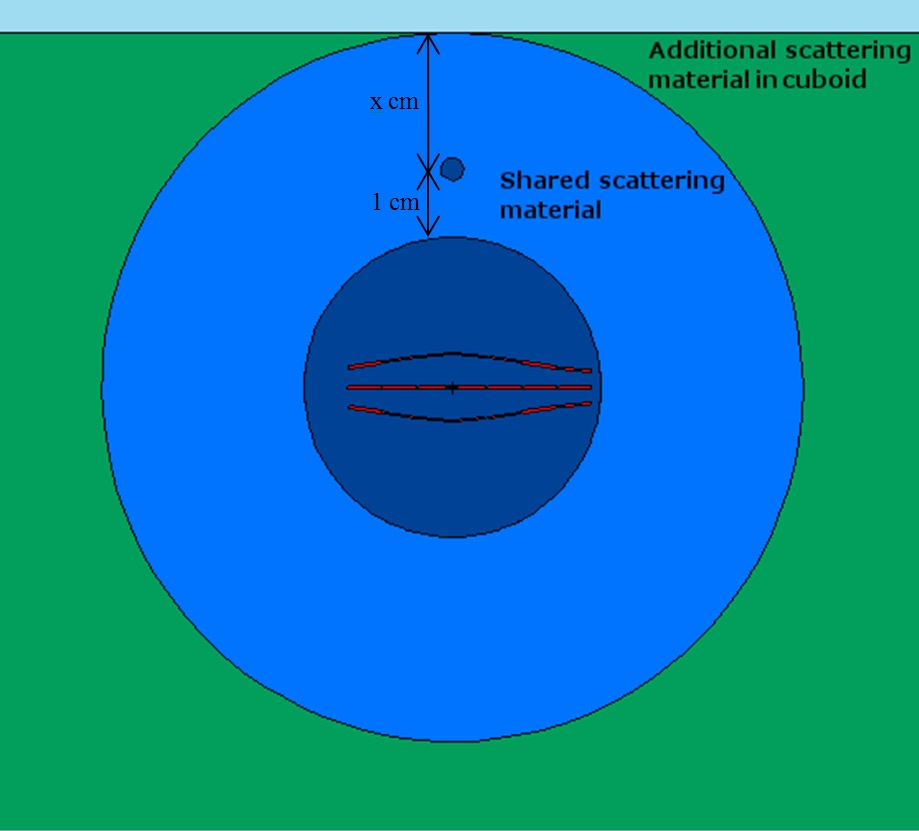
Visual representation of modeled phantom geometries. The outer blue circle represents the spherical phantom, while green square represents the cuboid phantom.

**Figure 7:**
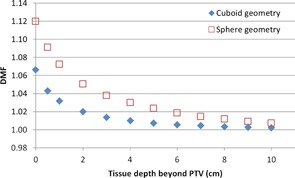
Contura modeled in different water phantoms.

To find the maximum DMF a plan could have, and thus the maximum deviation between planned and delivered dose, the Contura model was simulated in a spherical and cuboid water phantom. The spherical phantom was used to simulate a worst‐case scenario geometrically, as the average patient breast contour would not be a perfect sphere. A visualization of these phantoms can be seen in Fig. 6. The Contura model and reference point are shown in navy blue, the spherical water phantom is light blue, and the cuboid phantom is green, extending laterally past the edges of the figure. The sky blue color above the phantoms is air. The annotation “x cm” is the tissue depth, which varied from 0 to 10 cm. The results of this study are shown in Fig. 7.

The large differences in DMF in Fig. 7 demonstrate that the water phantom geometry used in the simulation is significant. A spherical water phantom yields higher values of DMF than a cuboid phantom. This could be due to the additional total scattering volume present in the cuboid phantom simulation. Figure 7 also indicates that, for a situation where no backscatter is present beyond the PTV, the TPS would produce a plan that would underdose the reference point by 7%–12%. As stated previously, the 12% DMF for the spherical phantom is the geometric worst‐case scenario, and the average patient breast would fall in between the two geometry measurements.

## IV. CONCLUSIONS

The amount of asymmetry achievable while still satisfying dose criteria in the Contura applicator was calculated, along with the associated V150 and V200 dose parameters (≤50cc and ≤10cc, respectively). The amount of asymmetry in Contura was found to be 14.7%. In comparison, the MammoSite ML and the SAVI had been found to be 12.2% and 37.3%, respectively. As one would expect, based on their construction, the capabilities of the Contura were closer to that of the MammoSite ML than to the SAVI. MCNP5 was then used to calculate the effect of not using inhomogeneity corrections, specifically for the case of the Contura balloon. The lack of tissue inhomogeneity correction in treatment planning systems can produce plans that underdose the PTV by up to 7%‐12% in the case where there is no backscatter past the PTV. In a radiation course like APBI where the total dose is lower compared to external beam treatments, this underdosing could be significant. To study the effect of dose asymmetry on the DMF, simulations of a highly asymmetric plan were compared to that of a symmetric plan, both using the Contura MC model. The asymmetric plan, with a dose distribution designed to reduce the skin dose, resulted in a DMF that did not differ in any statistically significant way to that of the symmetric case. Thus, the implementation of asymmetric dose distributions does not add any additional error to the dose near the skin.

APBI has gained in popularity and will likely be used more frequently in the future. Clinical studies have shown that it is a viable treatment option for those who qualify.^(1)^ These results reiterate the conclusions recently made by AAPM Task Group 186, that heterogeneity corrections be implemented clinically.
